# Humans inherit artificial intelligence biases

**DOI:** 10.1038/s41598-023-42384-8

**Published:** 2023-10-03

**Authors:** Lucía Vicente, Helena Matute

**Affiliations:** Department of Psychology, Deusto University, Avenida Universidades 24, 48007 Bilbao, Spain

**Keywords:** Psychology, Engineering

## Abstract

Artificial intelligence recommendations are sometimes erroneous and biased. In our research, we hypothesized that people who perform a (simulated) medical diagnostic task assisted by a biased AI system will reproduce the model's bias in their own decisions, even when they move to a context without AI support. In three experiments, participants completed a medical-themed classification task with or without the help of a biased AI system. The biased recommendations by the AI influenced participants' decisions. Moreover, when those participants, assisted by the AI, moved on to perform the task without assistance, they made the same errors as the AI had made during the previous phase. Thus, participants' responses mimicked AI bias even when the AI was no longer making suggestions. These results provide evidence of human inheritance of AI bias.

## Introduction

Over the last decades, the number of tools based on artificial intelligence (AI) designed to assist human decisions has increased in many professional fields such as justice^1,2^, personnel selection^3,4^ and healthcare^5–8^. In the medical realm, the advent of AI-based decision support systems has been welcomed as a promise to minimize errors in human decision-making^9–13^. Such optimism is founded on the impressive precision that artificial intelligence has reached in some clinical tasks such as image-based diagnostics, predicting the outcomes of interventions, or recommending treatments^13–16^. Thus, AI tools can help health professionals in, for example, diagnosis and triage tasks^17^ by offering them specific and quite accurate data-driven recommendations^18,19^. This cooperation between natural and artificial intelligence is hoped to augment clinicians' knowledge and capabilities, as well as compensate for some of the weaknesses of their human minds, such as cognitive biases^20–23^ and vulnerability to fatigue^24,25^, and therefore reduce diagnostic errors, improving clinical decisions^26^. But these AI tools are not free of errors and biases themselves.

Despite their promising benefits, the introduction of AI-based decision support systems in clinical contexts has also raised concerns about the peril of using biased AI to assist medical decisions^27–29^. People tend to perceive artificial intelligence algorithms as objective, secure^30^ and impartial^31^, but AI algorithms are a product of human design, so they often inherit our mistakes and biases^32,33^. Although AI-based tools have proven to be quite accurate, their performance is far from perfect and most of them still need to be validated in real-world settings^7^. Thus, while AI can help to overcome some of the limitations of human reasoning, other new problems may arise in the human-AI interaction^34^. Biased AI systems could sometimes diminish rather than augment the correctness of clinical decisions in that collaborative AI-human decision-making^35,36^.

Bias in an algorithm is defined as a systematic error in its outputs or processes^37,38^. One important potential source of bias in AI systems is biases or imbalances in the data used to train the algorithms^28,38–40^. AI algorithms identify patterns and generate predictions based on historical data (i.e. what happened in the past). High-quality data sets used to feed the algorithms are difficult to obtain^5^, particularly when they pertain to clinical data^41^. Consequently, if a given data set contains imbalances or biases^42^, the AI systems trained with these data will learn and potentially reproduce those biases^43^.

In the healthcare area, the data used to train the algorithms are usually the product of past human decisions, for example, medical decisions about what kind of patient requires a certain test or what kind of patient would benefit more from a given treatment. When the historical record of these choices shows an extended systematic error such as, for instance, a misdiagnosis of a certain pathology when certain features are present, the AI system trained with such historical data will simply inherit this bias^44^. It is relevant to note the technical nature of AI bias, as it is, in essence, a mathematical or statistical artefact. In an elementary form, this systematic error could be exemplified by a consistent misidentification of a certain pattern of coloured pixels in an image. However, this bias would reach serious implications if such an error in the model is not detected and the AI system is applied to decision-making in high-stakes scenarios, such as AI-based facial recognition systems^45^ to identify criminal suspects or AI-based image diagnosis^46^.

As a consequence, AI bias can also result in discrimination or prejudice towards a person or group of people^47^ since the patterns embedded in the historical data used to train algorithms often reflect systematic social and economic inequities. When an artificial intelligence system is trained with data that does not represent the diversity of the reality and population groups to which an AI tool is to be applied^42^, the model will produce undesirable effects due to the difficulty in generalising its predictions to social groups or environments with characteristics underrepresented in those databases^45,46,48^.

In high-stakes areas such as healthcare, clinicians assume the responsibility and accountability for the decisions of the AI-human team^49^. Thus, they are expected to supervise the outcomes of their artificial counterparts. Since there is a risk of bias (i.e., systematic errors) in the recommendations of AI-based decision support systems, health professionals should interpret AI advice as just an additional piece of evidence to help them in the decision-making process. Hence, they need to critically supervise and decide whether the AI advice is correct or useful for each decision^50,51^.

Some evidence has pointed out that this effective oversight and control of AI by humans could be possible^52–54^, while another corpus of research has documented an excessive trust towards AI recommendations, which calls into question the ability of humans to exercise a good supervision of algorithmic outcomes^55–57^. The tendency to over-rely on artificial intelligence can lead to humans uncritically adhering to AI recommendations, even incorrect ones^50,58,59^.

Human over-reliance on AI advice seems to be modulated by the context and the characteristics of the task (e.g., subjective vs objective) in which AI and humans collaborate^60–64^. In the clinical context, algorithmic advice is usually seen as trustworthy because artificial intelligence is perceived as accurate in objective and analytical tasks^55,62,65^, and these are the ones that are common in the medical domain, such as diagnosis and image classification. Thus, there are reasons to believe that decision-making in a health context could be particularly vulnerable to human over-reliance on AI advice, and therefore humans could tend to accept the recommendations of AI algorithms even when they are noticeably biased or erroneous.

There is some evidence that incorrect AI recommendations can have a detrimental influence on clinicians ' decision-making^66–69^. As an example, a recent study showed that when prescribing antidepressants in different scenarios, incorrect AI recommendations led to a reduction in the accuracy of the clinician's decisions in comparison to a baseline and a correct advice condition^70^. These results suggest that occasional mistakes in the AI recommendations can make people err, but our main concern refers to the presence of systematic biases in AI systems and to the potential of humans to perpetuate those biases. Thus, it is necessary to explore how humans react to systematic errors in AI advice, because AI bias may have a more profound impact on human behaviour than occasional and random errors. To our knowledge, very few investigations have addressed the effect of biased artificial intelligence systems on human behaviour^54^, and even fewer studies have focused, specifically, on the impact of algorithmic bias on human decisions in a clinical context.

The present research aims to answer two main questions. The first one is whether biased recommendations of an AI system can influence human behaviour, specifically decision-making, in a medical context. The recent study of Adam et al.^71^ represented an interesting starting point to this problem since they directly explored the influence of biased recommendations of an algorithm on the human response to mental health emergencies. These authors reported that the biased recommendations strongly influenced the decisions of experts and non-experts on how to respond to a crisis, while the decisions of participants without the advice of the algorithm were unbiased. In addition, we believe that to fully explore the potential impact of AI biases on human behaviour, it is also necessary to answer a second question. This question is whether, after the interaction with a biased AI system, people would reproduce those biases in their own decisions when they move on to a context without their artificial partner. The reader should consider the following scenario: given the case of a group of people accustomed to performing a specific task with the suggestions of a biased AI-based decision support system, is there a risk that the system's biased recommendations will exert a training effect such that people will reproduce the AI bias when making decisions on their own? Could the system influence the behaviour of these people so that they inherit the AI bias, even in a context without AI recommendations?

The potential inheritance of AI bias and its propagation through human decisions is a phenomenon that remains unexplored. The current research aims to examine how biased AI recommendations can influence people's decision-making in a health-related task and to test whether the impact of the biased advice on human behaviour extends beyond the phase in which the AI recommendations are explicitly present. That is, we will explore whether human decision-makers can inherit the bias of an artificial intelligence system.

## Overview of the experiments

In a series of three experiments, we empirically tested whether (a) people follow the biased recommendations offered by an AI system, even if this advice is noticeably erroneous (Experiment 1); (b) people who have performed a task assisted by the biased recommendations will reproduce the same type of errors than the system when they have to perform the same task without assistance, showing an inherited bias (Experiment 2); and (c) performing a task first without assistance will prevent people from following the biased recommendations of an AI and, thus, from committing the same errors, when they later perform the same task assisted by a biased AI system.

## Experiment 1

The first experiment tested the influence of explicitly biased recommendations made by a fictitious AI system on participants’ behaviour using a classification task with a medical-themed story: a simulation of an image-based diagnosis.

### Method

#### Ethics statement

The Ethical Review Board of the University of Deusto reviewed and approved the methodology reported in this article and the experiments were conducted according the approved guidelines. Informed consent was obtained from all participants. Due to ethical considerations, as well as to prevent the influence of prior knowledge and beliefs on experiments’ results, the clinical context, the classification task, the artificial intelligence system, its recommendations and its bias, the tissue sample images, the patients and syndromes used in these experiments, were all fictitious.

#### Participants

A group of 171 Psychology students took part in the experiment, but data from two of them were excluded following the data selection criteria described below. The final sample included 169 students (73.6% female, 20.7% male, 2.4% non-binary, mean age = 18.4, *SD* = 0.79). Their participation was anonymous and we did not ask for any personal data other than age and gender. Participants were randomly assigned to either one of two groups, assisted by AI (*n* = 85) and unassisted (*n* = 84). The post hoc sensitivity analysis showed that, with this sample size, we obtained a power of 0.80 to detect a size effect of *d* = 0.38 or higher in a test of difference between two independent means.

#### Materials and procedure

In the three experiments we used the same experimental task: a classification task framed in a fictitious health context. With this procedure we tried to simulate a clinical decision-making process that was assisted by a biased AI system for some participants, while others performed the task without assistance.

The experimental task was constructed though Qualtrics, a platform that also facilitated the data collection. We wanted to simulate a clinical decision-making process with a medical diagnosis task that was simple to learn, but also challenging enough to require sustained attention. With this aim, we used the stimuli created by Blanco et al.^72^ in their study related to causal illusion. Each stimulus consisted of a matrix of 50 × 50 pixels which represented a human tissue sample obtained from a given patient. Each tissue sample contained 2500 cells of two colours (dark pink and light yellow) randomly distributed in the matrix space so that there were no two identical samples. The proportion of dark and light cells in each tissue sample was variable, so we created a large set of different stimuli with varying levels of discrimination difficulty. The different proportions of dark and light cells for different stimuli were 80/20, 70/30, 60/40, 40/60, 30/70, and 20/80.

In the classification task, participants were instructed to observe a series of tissue samples, to decide, for each sample, whether it was affected or not by a fictitious disease called Lindsay Syndrome. Each tissue sample had cells of two colours, but one of them was presented in a greater proportion and volunteers were instructed to follow this criterion to identify the presence of the syndrome. A greater proportion of dark than light cells was described in the instructions as “Positive”, that is, affected by the Lindsay Syndrome. If the tissue sample had a greater proportion of light than dark cells, then it should be classified as a “Negative” because it was not affected by the syndrome. It is important to note that the assignment of dark and light colours to the positive/negative categories was randomly decided for each participant. For simplicity, in this section we describe only the Instructional Assignment A, but the reader should bear in mind that half of the participants received the opposite assignment.

To ensure that all participants correctly understood the instructions the experiment begun with a practice phase in which participants categorised six tissue samples with different dark/light colours proportions. Each sample was presented twice, so that the practice phase consisted of two blocks of six stimuli, presented one per trial, that is 12 trials in total. In the first block, the six samples were presented in order of difficulty. In the second block, the same six samples were presented in random order. If participants did not get five correct classifications out of six trials in the second block, they had to repeat all the practice phase.

Once the volunteers finished the practice phase, they were randomly distributed into two groups, AI-assisted and unassisted. The design of the three experiments is summarized in Table 1.

**Table 1:** Design summary of the three experiments. *n* is the number of final participants in each group. The two columns labelled Trials indicate the number of tissue sample images used in Phase 1 and Phase 2 of the classification task. As participants viewed one image per trial, i.e., each tissue sample was shown on a separate screen, the total number of images equals the total number of trials for each phase.

Experiment	Group	*n*	Classification Task			
			Phase 1	Trials	Phase 2	Trials
Experiment 1	Assisted	85	AI-assisted	60	–	–
	Unassisted	84	Unassisted		–	
Experiment 2	Assisted	100	AI-assisted	60	Unassisted	25
	Unassisted	99	Unassisted		Unassisted	
Experiment 3	Assisted-Unassisted	98	AI-assisted	40	Unassisted	40
	Unassisted-Assisted	99	Unassisted		AI-assisted	

**Figure 1:**
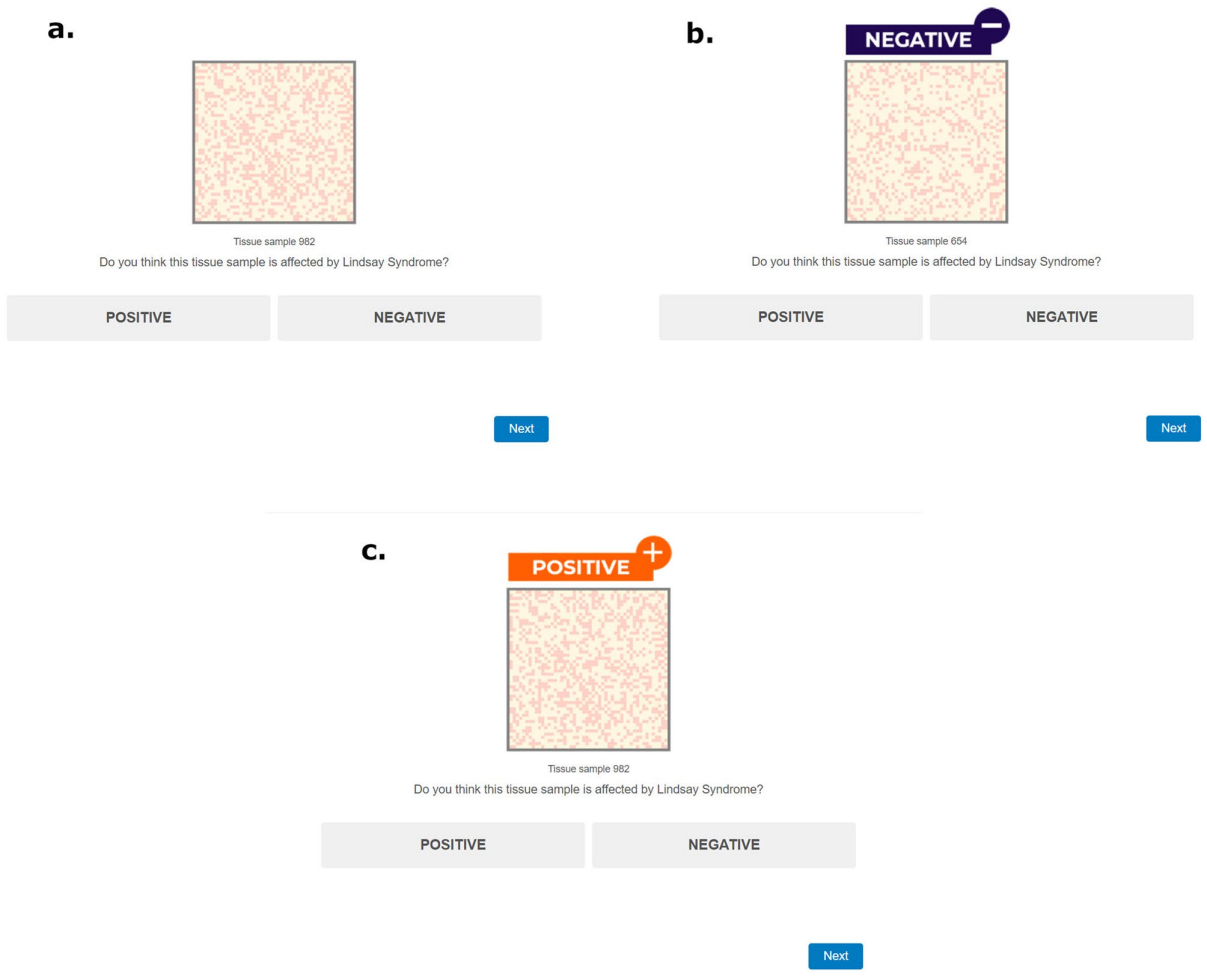
Screenshots showing examples of trials in the unassisted and AI-assisted groups. (**a**) depicts a 40/60 unassisted trial, (**b**) exemplifies an AI-assisted 30/70 trial where the AI made a correct recommendation (following the Instructional Assignment A, a greater proportion of light-coloured cells should be classified as a negative case). At the bottom of the image, (**c**) illustrates a biased AI recommendation for a 40/60 trial that should be negative following Instructional Assignment A, but the AI labelled it as positive. Stimuli were adapted from “Are the symptoms really remitting? How the subjective interpretation of outcomes can produce an illusion of causality” by Blanco, F., Moreno-Fernández, M. M., and Matute, H. *Judgment and Decision Making*, 15, pp. 575 (2020). CC BY 3.0.

The aim of the participants was to classify a series of tissue samples following the criteria they had just learned in the practice phase. Phase 1 of this experiment comprised 60 tissue samples (i.e., trials). In the AI-assisted group, the simulated tissue sample and the AI recommendation were presented simultaneously in each trial. The recommendation had the form of an orange label with the text "POSITIVE + ”, or a blue label with the text "NEGATIVE—", placed above the tissue sample. In the unassisted group, only the tissue sample was presented in each trial. Both groups viewed the same stimuli, the only difference between them was the presence or absence of the advice of the fictitious AI. The sequence of trials included ten stimuli (i.e., tissue samples) of each of the dark/light cell proportions, that is, 80/20, 70/30, 60/40, 40/60, 30/70 and 20/80, resulting in a total of 60 stimuli. Figure 1 shows some examples of trials in both the unassisted and the AI-assisted groups. The simulated AI of our experiment made correct recommendations on 50 tissue samples out of 60. Thus, our hypothetical model correctly classified approximately 80% of the tissue samples during Phase 1. However, this simulated AI model showed a bias or systematic error: the recommendations for the ten stimuli with the dark/light cells ratio of 40/60 were always wrong. In these samples, there was a contradiction between the evidence (i.e., number of dark/light cells in the tissue) and the recommendation given by the AI (i.e., the blue or orange labels with the negative or positive suggestions). For example, following the instructions, the 40/60 tissue samples had a greater number of light cells so they should be classified as negative, however, the recommendation given by the AI for the 40/60 samples was positive.

The order of the stimuli was randomly assigned through the sequence of 60 trials, with one exception: the 40/60 stimuli, where the AI recommendation was always erroneous, did not appear during the first 10 trials of the sequence of 60 trials. This manipulation tried to keep certain level of trust in the AI model before the problematic stimuli showed up. We wanted the AI bias not to be so evident from the beginning of the task. The ten 40/60 stimuli did appear in the sequence of the next 50 trials, randomly intermixed with the stimulus of other proportions.

After the classification task was completed, we asked participants, as a manipulation check, to indicate whether the AI had offered any recommendations to them during the task. Next, volunteers answered a series of questions about their own performance and about how they had perceived the AI’s performance during the classification task. Participants indicated on a Likert scale from 1 (*not at all*) to 9 (*completely*) their level of trust on the artificial intelligence used in this experiment, and their trust in artificial intelligence applied to healthcare, in general.

The main dependent variable in our experiment was the number of misclassifications of the ten 40/60 tissue samples. Although the stimulus 40/60 was more difficult to classify than other stimuli, its discrimination was clear, and it was possible to detect the AI mistakes easily. Thus, we expected the unassisted group to classify them correctly. However, since the AI showed a rather high reliability, we expected volunteers in the group assisted by the biased AI to get used to performing the task following the AI recommendations and without careful examination of the tissue sample. As a result, the AI-assisted group would misclassify more often the 40/60 stimuli, where the recommendations were erroneous, than the unassisted group.

#### Data selection criteria

If participants failed to reach the threshold of five correct classifications out of six trials in the second attempt of the practice phase, their data were excluded from the analyses. In addition, data from participants who misclassified tissue samples on more than half of the trials of the first phase of the classification task (i.e., more than 30 trials out of 60), were excluded from the analyses, as they were, presumably, paying little attention.

### Results and discussion

**Figure 2:**
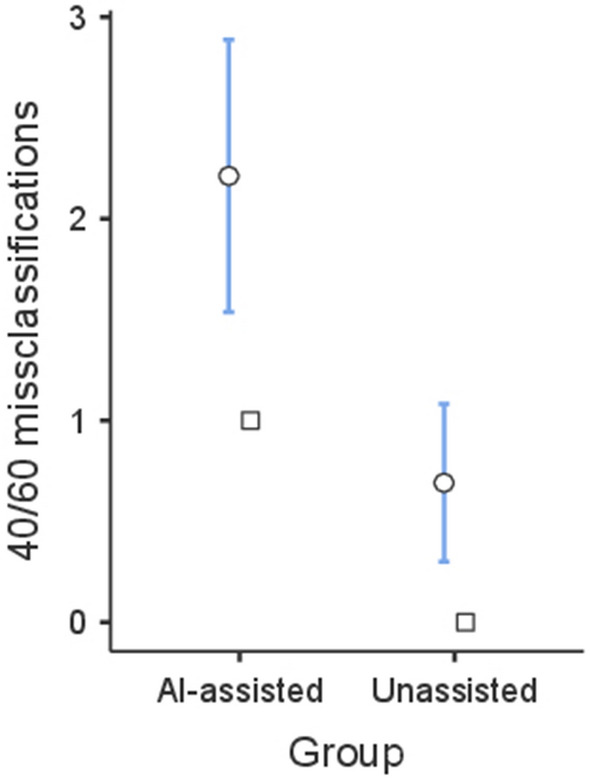
Mean number of misclassifications of the ten 40/60 tissue samples in the AI-assisted group (n = 85) and the unassisted group (n = 84) in Experiment 1. Error bars represent 95% CI.

As expected, participants who performed the task assisted by a biased AI made more errors than unassisted participants (see Fig. 2). The mean number of misclassifications of the 40/60 trials was 2.21 (*SD* = 3.17) in group AI-assisted, and 0.69 (*SD* = 1.83) in the unassisted group. This difference was significant, *t(*167) = 3.81, *p* < 0.001, *d* = 0.586. These results show that the explicit recommendations of a biased AI influenced participants’ behaviour and increased the number of errors in a health framed task.

On average, participants though they had performed the task fairly well and showed a moderate trust in AI in healthcare. In the AI-assisted group we found a positive correlation between participants’ incorrect classifications of the 40/60 samples and how helpful they considered the AI of the experiment to be, *r* = 0.544, *p* < 0.001, the accuracy they attributed to the AI recommendations during the experiment, *r* = 0.563, *p* < 0.001, and their confidence in AI in the health domain in general, *r* = 0.470, *p* < 0.001.

These results evidenced human compliance with the AI recommendations during a decision-making process. A large part of participants from the AI-assisted group reproduced the AI bias in their own responses to the medical task.

## Experiment 2

The purpose of Experiment 2 was (a) to replicate the observation of Experiment 1 that a biased AI can influence human decision-making, (b) to test whether such influence persists when the AI is no longer present, and (c) to test whether this influence generalizes to novel stimuli as well. Additionally, we now also measured and analysed changes in the participants' behaviour over the sequence of trials during the classification task.

### Method

#### Participants

The final sample included 199 participants (65.3% female, 30.7% male, 4% non-binary, mean age = 26.2, *SD* = 6.95). We initially recruited 200 participants through Prolific Academic, but data from one participant was excluded due to the data exclusion criteria described in Experiment 1. Participation in the experiment was offered only to those applicants in Prolific Academic's pool who speak English fluently and had not participated in previous experiments from our research team. Volunteers were randomly distributed between two groups, AI-assisted (*n* = 100) and unassisted (*n* = 99). A sensitivity analysis showed that, with this sample size, we obtained a power of 0.80 to detect a small-sized effect (*f* = 0.09) for a repeated measures ANOVA with within-between interaction.

#### Materials and procedure

The procedure was similar to that of Experiment 1. The practice phase and the classification task with 60 trials were identical to those of the previous experiment. The only difference during the 60 trials phase was that in Experiment 2 the order of appearance of each stimulus was randomly assigned to a fixed position in the 60-trial sequence of the task. The creation of this fixed randomised sequence ensured that all participants viewed each stimulus in the same position. This facilitated the measurement of changes in the behaviour of both groups and their comparison, with particular attention to participants' responses in those trials where the AI recommendation was erroneous.

The main novelty in Experiment 2, with respect to Experiment 1, was the addition of a second phase of 25 trials in which both groups had to sort the tissue samples without assistance (see Table 1). That is, participants in the unassisted group continued performing the task as in the previous phase, while AI-assisted participants switched to performing the task without assistance. An additional feature of this second phase was the introduction of five trials of novel and ambiguous stimuli with a dark/light cell ratio of 50/50, so that it was not possible to assign these stimuli to either one of the two categories, positive or negative, based on the instructions received or on specific recommendations of the AI during the previous phase. Thus, for this second phase, we expected that participants in the AI-assisted group would tend to classify both the 40/60 and the 50/50 samples in the same category as the AI biased recommendation suggested for the 40/60 stimuli in the previous phase. By contrast, we expected participants in the non-assisted group to classify correctly the 40/60 stimuli and to classify the 50/50 ambiguous stimuli randomly. We would interpret these results as an inheritance and a generalization of the AI bias. Thereby, the purpose of this experiment is not only to reproduce but also to extend the results of Experiment 1, showing that biased AI recommendations may influence participants' behaviour even when the AI is no longer present and even in novel conditions.

Also, in this phase, stimuli with the dark/light cell ratio 80/20 and 20/80 were not included in the classification task, because we considered them too easy and we were mainly interested in participants' classification of the 40/60 and 50/50 stimuli. Thus, the second phase of the task consisted on five stimuli of each of the 70/30, 60/40, 50/50, 40/60, and 30/70 proportions, resulting in 25 trials. The order of appearance of each trial was randomly assigned to a fixed position in the 25-trial sequence of the task. We measured an additional dependent variable in this phase: the number of times each participant classified the five 50/50 stimuli in the same direction as the bias of the AI recommendations. We named this variable as biased classifications.

In addition, in Experiment 2 we intended to measure and analyse within-subject changes in participants' behaviour throughout Phase 1 and Phase 2 of the classification task. In order to analyse those changes in the participants' classification errors during Phase 1, we divided the ten trials with the 40/60 tissue samples into two blocks of five trials each. Thus, the first five 40/60 trials of Phase 1 constituted Block 1, and the second five 40/60 trials formed Block 2. These smaller units allow us to observe the trends followed by participants’ misclassification errors. Furthermore, as Phase 2 comprised five additional 40/60-stimuli trials, these five trials formed Block 3 for the purpose of analyses, so the division of two blocks in Phase 1 also helped to create three equivalent units to compare the evolution of the groups' responses during the experiment. At the end of the classification task, participants answered the same post-experimental questions as in Experiment 1. In the present Experiment 2, we added two questions to ask volunteers directly whether they based their answers on the AI recommendations and whether they detected any errors in the AI recommendations. Experiment 2 was preregistered in https://aspredicted.org/3a9wj.pdf.

### Results and discussion

**Figure 3:**
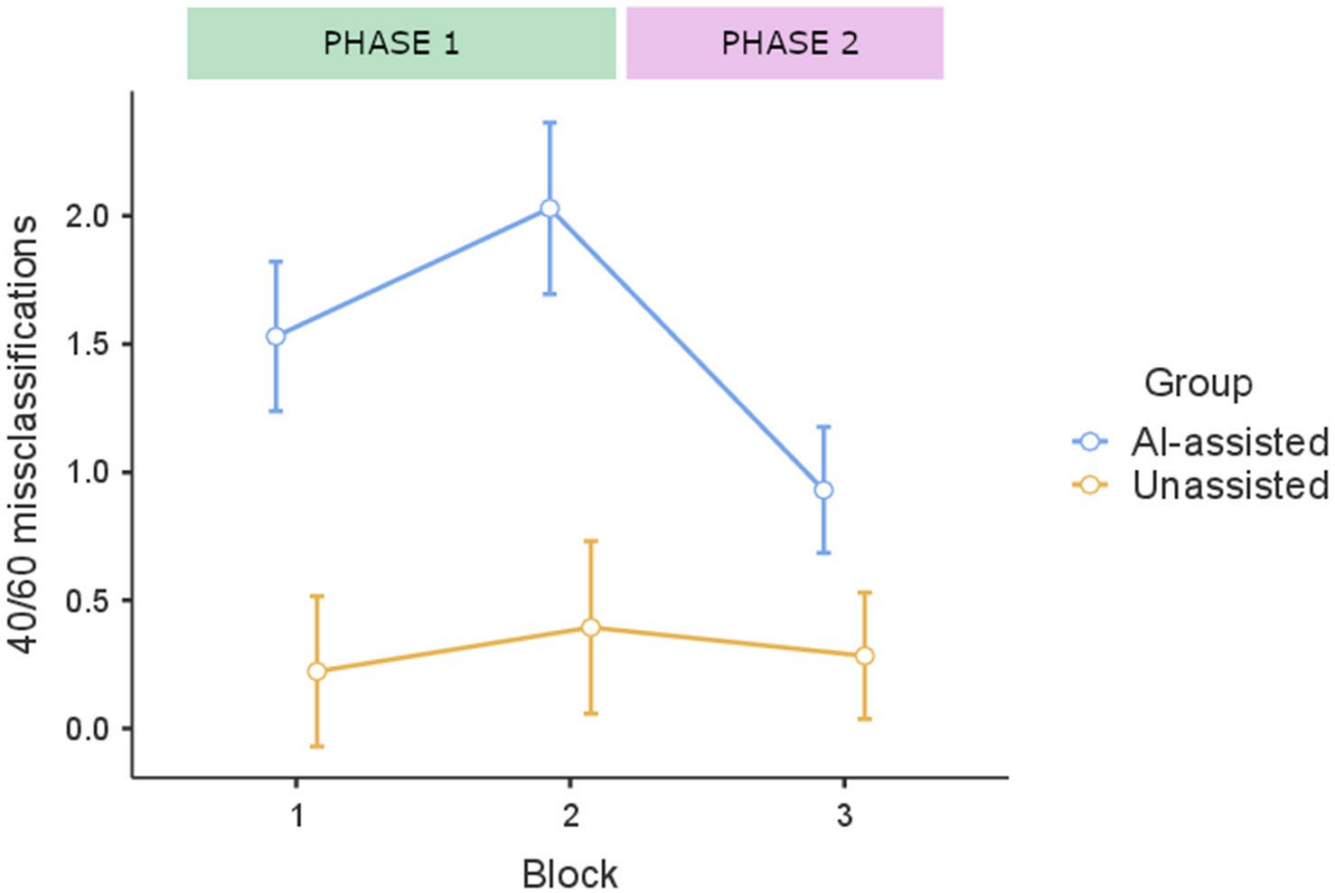
Mean number of misclassifications on the three blocks of 40/60 trials in the AI-assisted group (n = 100) and the unassisted group (n = 99) along the two phases of Experiment 2. Blocks 1, 2 and 3 comprise five 40/60 trials each. Error bars represent 95% CI.

As expected, the AI-assisted group made more errors in the classification of the 40/60 stimuli in both phases of the classification task, than the unassisted group. Importantly, even in Phase 2, that both groups performed without the support of AI recommendations, the AI-assisted group committed significantly more misclassifications than the unassisted group (see Fig. 3). These impressions were confirmed by a mixed ANOVA 3 (Block) × 2 (Group) that showed a main effect of Group, *F* (1,197) = 41.3, *p* < 0.001, *η*^*2*^_*p*_ = 0.173, a main effect of Block, *F* (2,394) = 25.3,* p* < 0.001, *η*^*2*^_*p*_ = 0.114, and a Group x Block, interaction* F* (2,394) = 17.4, *p* < 0.001,* η*^*2*^_*p*_ = 0.081.

Within group *post-hoc* comparisons, with Tukey correction, showed an increase in the 40/60 misclassifications in the AI-assisted group between Block 1 and Block 2, *t(*197) = − 4.657, *p*_*t*_ < 0.001, as well as a decrease between Block 2 and Block 3, in which this group no longer had the AI recommendations, *t*(197) = 8.87, *p*_*t*_ < 0.001. In this last phase, although none of the two groups were assisted by the biased AI, there was still a significant difference between them *t*(197) = 3.66, *p*_*t*_ = 0.004. That is, the AI-assisted group did not reduce the number of errors enough to reach the performance of the non-assisted group.

One of the main novelties of the second phase of Experiment 2 was the addition of five tissue samples with a 50/50 dark/light cell proportion. Since neither of the groups received instructions or AI recommendations on how to classify these stimuli, we expected the AI-assisted group to classify the ambiguous novel 50/50 stimuli in the same direction as the AI bias from the previous phase. An independent samples t-test showed that the AI-assisted group made more biased classifications of the 50/50 samples, *M* = 2.91*, SD* = 2.13, than the unassisted group, *M* = 2.15*, SD* = 2.02, with a small but significant difference between both groups, *t(*197) = 2.58, *p* = 0.011, *d* = 0.366. This suggest that the inherited bias is not constrained to the stimuli where the AI made errors, but can also generalize to novel stimuli that had not been seen before and had not received any previous AI recommendation.

Regarding the post-experimental questions, 58% of participants from the AI-assisted group detected errors in the IA advice. Moreover, similar to Experiment 1, participants who made more errors in the classification task also found the AI of our experiment more helpful, *r* = 0.638, *p* < 0.001, perceived it to be more accurate, *r* = 0.612, *p* < 0.001, and trusted more, in general, in the usefulness of AI in the health context, *r* = 0.492, *p* < 0.001.

## Experiment 3

Experiment 3 tried to replicate the bias inheritance effect observed in Experiment 2 for the IA-assisted group in the 40/60 and 50/50 stimuli during the non-assisted phase. In addition, we sought to extend the results of the previous experiments by analysing the effect of the order in which the AI-assisted phase takes place. We hypothesised that performing the classification task without assistance first, could have a protective effect against the biased recommendations when participants switched to performing the task assisted by the misleading AI.

### Method

#### Participants

A group of 197 participants (49.2% male, 47.7% female, 3% non-binary, mean age = 27.1, *SD* = 8.28) from Prolific Academic took part in the experiment (initially we recruited 200 participants but data from three of them were excluded following the data exclusion criteria described in Experiment 1. Participation in the experiment was offered only to those applicants in Prolific Academic's pool who speak English fluently and had not taken part in previous studies carried out by our research team. Volunteers were randomly assigned to groups AI-assisted → unassisted (*n* = 98) and unassisted → AI-assisted (*n* = 99). A sensitivity analysis showed that, with this sample size, we obtained a power of 0.80 to detect a small-sized effect (*f* = 0.08) for a repeated measures ANOVA with within-between interaction.

#### Materials and procedure

The main difference on the design of Experiment 3 with the previous experiments is that all participants went through both experimental conditions, that is assisted by the biased AI recommendations and unassisted, but in different order (see Table 1). Thus, the procedure of Experiment 3 was similar to that of the previous experiments, but with some modifications to adapt it to the design of the present experiment. Specifically, a change in the number of trials in Phase 1 and Phase 2, and some slight adjustments in the task instructions were necessary.

In Experiment 3, each of the two phases of the task had 40 trials. Table 2 depicts the number of trials of each type of stimuli for the AI-assisted and the unassisted phases of this experiment. The ten 40/60 stimuli appeared intermixed with stimuli of other proportions as in the previous experiments. The order of trials within each phase was random and identical for both groups, but in neither case did the 40/60 stimulus appear among the first five trials of the sequence. The unassisted phase of the task was also characterised by the inclusion of five ambiguous stimuli (see Table 2).

**Table 2:** Number of trials for each type of tissue sample of different dark/light cells proportions from the AI-assisted and unassisted phases of Experiment 3.

Phase	Stimuli							
	80/20	70/30	60/40	50/50	40/60	30/70	20/80	Total
*AI-assisted*	6	6	6	0	10	6	6	40 trials
*Unassisted*	5	5	5	5	10	5	5	40 trials

Given that all participants were assisted by the AI in one phase of this experiment, but were unassisted in another phase, the task instructions and screens explicitly informed participants when the AI system was connected and gave them recommendations, and when the AI was off, so that they had to perform the task without assistance. In Experiment 3 two images were included to emphasise this information.

Similar to Experiment 2, in Experiment 3 the ten 40/60 tissue samples from each phase were divided into two blocks to analyse within-subject changes in misclassification errors. Hence, the ten 40/60 trials from Phase 1 were divided into two blocks, Block 1 and 2, of five trials each. The same procedure was followed for the ten 40/60 trials from Phase 2, which were also divided into two blocks of five trials, Blocks 3 and 4. In total, four blocks of five 40/60 trials were created for analyses purposes.

The post-experimental questions in Experiment 3 were the same as in Experiment 2, but we made a modification to the question about whether the AI had been on and specified that we were referring to whether it had been connected at any point throughout the experiment. We thought that this specification was necessary because the group AI-assisted → unassisted might have interpreted that this question referred only to the last phase. Experiment 3 was preregistered in https://aspredicted.org/hq54a.pdf.

### Results and discussion

The group that completed the first phase (i.e., Blocks 1 and 2) assisted by the biased AI made more errors in the 40/60 trials than the unassisted group. In Experiment 3, as well as in Experiment 1 and Experiment 2, the participants assisted by the AI made errors specifically on 40/60 trials, while participants' misclassification rates for other stimulus classes were close to zero, as can be seen in Table 3. This confirms that the classification task was easy and that the biased AI recommendations were the factor that misled participants.

**Table 3:** Mean misclassification rates (and standard deviations) for all stimuli of different proportions of dark/light cells used in the classification task in the three experiments. The misclassification rate per stimulus class is the total number of classification errors made on stimuli of a given class divided by the total number of such stimuli in each phase of the experiment. For Experiment 3, the group column shows which phase of the task each group completed first, AI-assisted refers to the AI-assisted → unassisted group, and Unassisted is the Unassisted → AI-assisted group.

Stimuli	Group	Mean misclassification rate				
		Experiment 1	Experiment 2		Experiment 3	
			Phase 1	Phase 2	Phase 1	Phase 2
80/20	AI-assisted	0.00 (0.01)	0.00 (0.00)		0.00 (0.05)	0.00 (0.02)
	Unassisted	0.00 (0.01)	0.00 (0.00)		0.00 (0.00)	0.00 (0.00)
70/30	AI-assisted	0.00 (0.00)	0.00 (0.02)	0.01 (0.04)	0.00 (0.01)	0.00 (0.00)
	Unassisted	0.00 (0.02)	0.00 (0.22)	0.00 (0.02)	0.00 (0.02)	0.00 (0.03)
60/40	AI-assisted	0.01 (0.06)	0.00 (0.03)	0.02 (0.08)	0.01 (0.04)	0.01 (0.04)
	Unassisted	0.03 (0.11)	0.02 (0.07)	0.03 (0.10)	0.04 (0.11)	0.00 (0.03)
40/60	AI-assisted	0.19 (0.28)	0.35 (0.39)	0.18 (0.31)	0.37 (0.37)	0.33 (0.38)
	Unassisted	0.06 (0.16)	0.06 (0.15)	0.05 (0.15)	0.07 (0.18)	0.37 (0.41)
30/70	AI-assisted	0.00 (0.00)	0.00 (0.02)	0.02 (0.10)	0.01 (0.07)	0.05 (0.13)
	Unassisted	0.00 (0.04)	0.00 (0.03)	0.00 (0.03)	0.00 (0.02)	0.01 (0.07)
20/80	AI-assisted	0.00 (0.03)	0.00 (0.01)		0.00 (0.01)	0.00 (0.00)
	Unassisted	0.00 (0.01)	0.00 (0.01)		0.00 (0.00)	0.00 (0.00)

**Figure 4:**
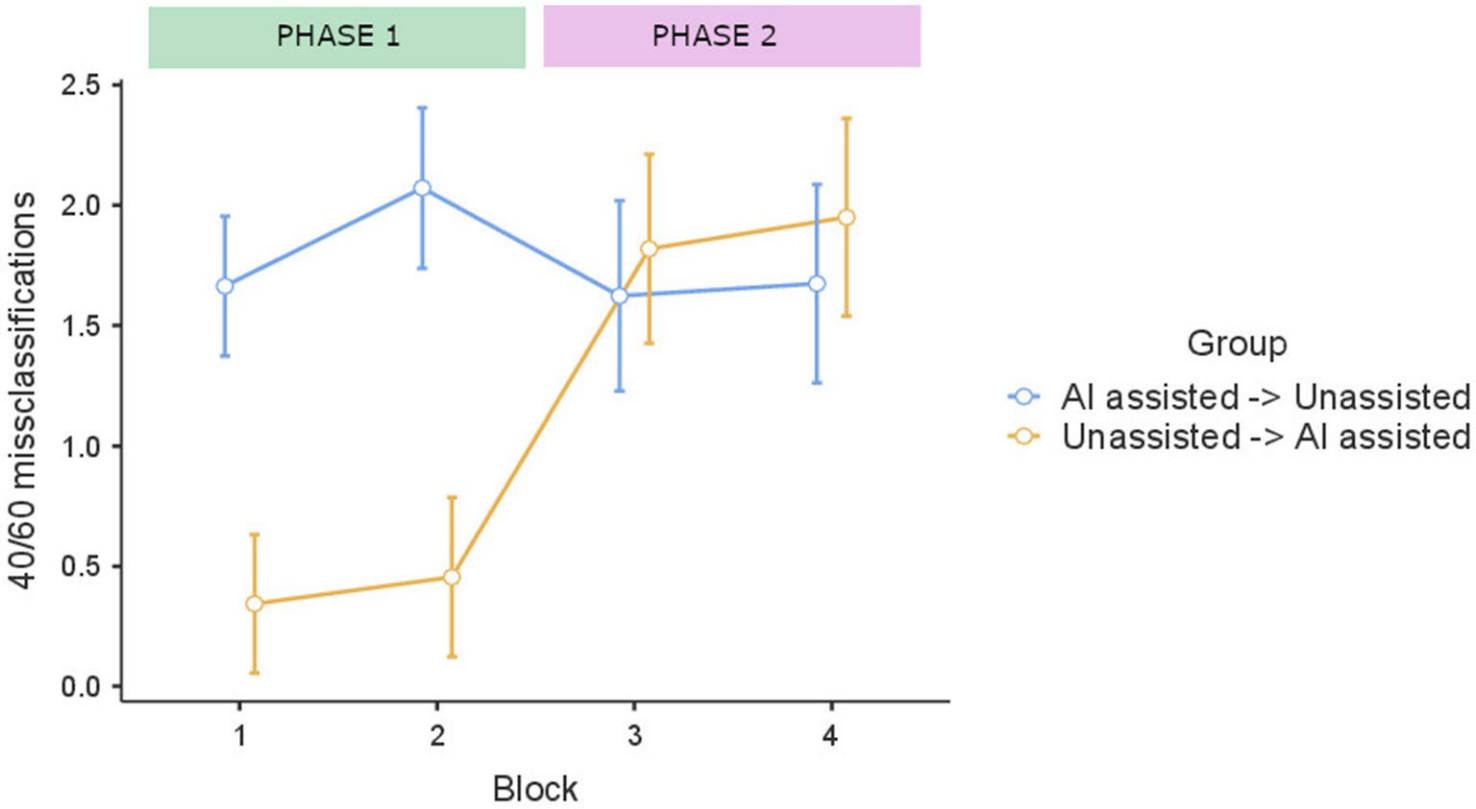
Mean number of misclassifications on the four blocks of 40/60 trials in the AI-assisted → unassisted group (n = 98) and the unassisted → AI-assisted group (n = 99) along the two phases of Experiment 3. Blocks 1, 2, 3 and 4 comprise five 40/60 trials each. Error bars represent 95% CI.

Interestingly, when there was a change in task conditions between Blocks 2 and 3, we observed an increase in 40/60 misclassifications for the unassisted → AI-assisted group, but did not observe an opposite trajectory for the AI-assisted → unassisted group. When this group transited from the AI-assisted to the unassisted phase, its mean number of errors in 40/60 trials was not reduced. During the second phase (i.e., Blocks 3 and 4) both groups committed a similar number of errors, although in this second phase the unassisted → AI-assisted group received biased recommendations from the AI and the AI-assisted → unassisted group did not. Thus, the group of participants who completed Blocks 1 and 2, assisted by the AI recommendations exhibited the same errors as the AI system when, during the second phase, in Blocks 3 and 4, they had to perform the task without guidance (see Fig. 4).

These impressions were confirmed by a mixed ANOVA 3 (Block) × 2 (Group) that showed a main effect of Group, *F (*1,195) = 7.59, *p* = 0.006, η^2^_*p*_ = 0.037, a main effect of Block *F (*1,585) = 26.0, *p* < 0.001, η^2^_*p*_ = 0.118, and a Group x Block interaction, *F (*1,585) = 43.8, *p* < 0.001, η^2^_*p*_ = 0.183. For the unassisted → AI-assisted group, *post-hoc* comparisons (Tukey correction) confirmed an increase in 40/60 errors between Block 2 and Block 3, *t(*195) = 8.75, *p* < 0.001, when this group switched to performing the task with the AI assistance. Conversely, for the AI-assisted → unassisted group an increase in 40/60 errors was detected between Block 1 and Block 2, *t(*195) = − 4.05, *p* = 0.002, during the AI-assisted phase of the task, but no differences were observed between the other blocks. So, there was not a significant decrease in the number of 40/60 misclassifications for this group when they switched from Block 2 to Block 3, that is, when they switched to the phase without AI recommendations, *t(*195) = 2.86, *p* = 0.085. This suggests that the responses of the AI-assisted → unassisted group reproduced the systematic errors of the AI recommendations during the unassisted phase, a result that supports the inheritance bias effect.

Similarly, the mean number of biased classifications of the 50/50 ambiguous stimuli during the unassisted phase in the AI-assisted → unassisted group was 3.72 (*SD* = 2.02), and 2.65 (*SD* = 1.88) in the unassisted → AI-assisted group. This difference was significant, *t(*195) =—4.28, *p* < 0.001, *d* =—0.609. Both results replicate those observed in Experiment 2, and add support to the human inheritance of AI bias effect.

It is important to note that in this experiment 80.7% of participants detected mistakes in the AI recommendations. Although participants found that the AI was not entirely accurate, many of them still relied on the AI recommendations to perform the task. In contrast to previous experiments, in this experiment we did not observe a positive correlation between the number of 40/60 misclassifications and how much participants considered AI to be helpful, *r* = 0.051, *p* = 0.479, accurate, *r* = 0.055, *p* = 0.442, and reliable in the health domain,* r* = 0.083, *p* = 0.246. Thus, in this experiment, participants' responses seemed to be less influenced by their prior trust in AI.

## General discussion

Our results show that biased recommendations made by AI systems can adversely impact human decisions in professional fields such as healthcare. Moreover, they also show that such biased recommendations can influence human behaviour in the long term. Humans reproduce the same biases displayed by the AI, even time after the end of their collaboration with the biased system, and in response to novel stimuli. Although this inheritance bias effect could have serious implications, to the best of our knowledge it had not yet been explored in any empirical research.

Intending to empirically test the potential effect of the human inheritance of the AI bias, we conducted three experiments. As we had hypothesized, the AI-assisted participants made more errors than the unassisted participants in the classification task, both during the AI-aided and non-aided phases, a result consistently observed over the three experiments. These results are robust: we obtained the same effect in a laboratory experiment conducted under controlled conditions with a sample of university students (Experiment 1) and in two online experiments with an international sample recruited via Prolific Academic (Experiments 2 and 3).

Importantly, Experiments 2 and 3 evidence that participants from the AI-assisted group still misclassified, specifically, the 40/60 stimulus in the non-aided phase of the experiment, meaning that their mistakes when performing the classification task by themselves mimicked the bias previously shown by the AI during the first phase. Moreover, we also observed a tendency in the AI-assisted group to categorize the new 50/50 stimuli during the unassisted phase in the same direction as the AI misclassified the 40/60 stimuli during the previous phase. We interpret that participants used the AI biased recommendations as a cue to categorise ambiguous stimuli, which could not be classified according to the classification criteria specified in the instructions. This suggests that participants did not only inherit the AI bias, but also generalized it to novel ambiguous stimuli.

In sum, we observed an inheritance effect of the AI biased recommendations from the first phase of the experiment on the participants' responses during the second phase, where they were no longer supported by the AI. We believe that this is the most interesting and novel result of our present work because it shows an influence of AI bias on human decisions that extends beyond the stage and stimuli in which the AI recommendations are explicitly present. Thus, AI biases could have the potential to propagate through humanity. To our knowledge, this inheritance of the bias effect has not been previously revealed in any empirical research.

The presence of AI erroneous recommendations for the 40/60 samples resulted in the AI-assisted group misclassifying significantly more 40/60-samples than the group unassisted. In Experiments 2 and 3, we also analysed changes in participants' behaviour over the sequence of trials, and observed an increase in the number of 40/60 misclassifications throughout the AI-assisted phase. This result suggests that participants’ monitoring of the information decreased and the tendency to rely on the AI recommendations to make their judgment increased as their experience with the AI increased. This could be due to fatigue but also to habituation and increased trust in the AI recommendations.

The “Negative” or “Positive” recommendation labels, in blue and orange colours, were probably more salient to participants' attention than the tissue samples that they had to classify, which were, in comparison, more complex. Thus, the tissue samples required effortful observation and processing to make a correct judgment about the presence (i.e., positive) or absence (i.e., negative) of the syndrome. Recommendation labels provided by the AI could have interfered with the participants' assessment of tissue sample objective information^73^. Volunteers may also have been reluctant to engage in a deep assessment of the reliability of each AI recommendation^74^ and, instead, they probably developed general rules about whether or not to follow AI suggestions, what diminished their ability to detect and correct the biased advice^75^.

During the unassisted phase of the task, we detected a reduction in the errors made by the AI-assisted group, but they still misclassified the 40/60 stimuli more than the unassisted group. This effect could have two possible interpretations: (a) a difficulty for participants in the AI-assisted group to regain control over the task because they had to change to slower and more conscious processing^76^ when they no longer had AI support^77^ or (b) a training effect of the recommendations made by the AI on our participants' responses so that they learnt from the AI bias.

Regarding our first interpretation, if participants trusted AI recommendations to the detriment of analytic or effortful processing^34,74^ during Phase 1, then when they moved to Phase 2, and they needed to take complete control of the task, they would have needed some time to adjust to the new task demands and redirect attentional resources. It seems that, the transition from automated to controlled performance was slow, and participants were still heavily influenced by AI bias. The errors derived from this slow transition had no serious consequences in our experiments, but there are professional areas, such as healthcare, where the consequences of decisions taken under the influence of an inherited bias could be fatal.

Concerning the second interpretation stated above, in Experiment 3, we did not observe a decrease in the errors made by the AI-assisted group in the transition from the AI-assisted to the unassisted phase. Maybe AI recommendations modelled participants' behaviours so that they learnt a new classification criterion based on the biased AI output. This could explain why the mean number of 40/60 misclassifications remained high in the context without the AI for the participants who had previously been supported by the biased AI in Experiment 3. If errors in the unassisted phase of the task were only due to difficulty in regaining control over the information processing, these errors should have been progressively reduced throughout the successive trials of this phase. Moreover, the fact that the inherited bias generalized to the ambiguous 50/50 stimuli also points to a training effect of the biased AI model on human participants.

The results of our three experiments support a human over-reliance on the recommendations of AI systems^70,71^, and add the new finding of the inherited bias effect. Human trust in automation influences the tendency to over-accept algorithmic outcomes^58,59^ even when they are noticeably wrong. This means that humans are not only willing to rely on AI because they are “cognitive misers”, that take mental shortcuts when making decisions, but also because they perceive artificial intelligence to be trustworthy^65,78^. It has been suggested that trust could induce compliance with AI advice due to an authority effect^79^. In Experiments 1 and 2, we detected that participants who perceived the AI of the experiment as more helpful and accurate, and trusted more in the usefulness of artificial intelligence in healthcare in general, were those who followed the AI recommendations more often, and committed more errors in the classifications task as revealed by the positive and significative correlations observed between the mean number of 40/60 misclassifications in the task and the participants' answers to the post-experimental questions.

As a limitation to our present work, it could be argued that our experimental task was a simulation of clinical decision-making with a fictitious diagnostic process and a fictitious artificial intelligence system. Although our experiments simplify a potential real-world setting, we believe that our controlled experimental task can help to analyse which basic psychological processes mediate on human-AI collaboration. We created a classification task with a low level of uncertainty, in which participants were provided with a clear classification criterion to perform the task, in which the error in the recommendations was systematic, controlled, and noticeable by the control participants, and in which prior expertise had no influence. These characteristics make our experimental task a fruitful method to study human reliance on AI algorithms and the potential inheritance of their biases.

## Data Availability

The data and materials for this experiment are freely available on the Open Science Framework at https://osf.io/ukehm/. Experiments 2 and 3 were preregistered.
